# Unusual Manifestation of Superior Mesenteric Artery Syndrome in a Female Patient: A Case Presentation

**DOI:** 10.7759/cureus.800

**Published:** 2016-09-22

**Authors:** Christian Fisahn, Marc Moisi, Randle Umeh, Scott Sylvester, Prasanthi Maddali, Susan J Tubbs, Rod J Oskouian, Marios Loukas, R. Shane Tubbs

**Affiliations:** 1 Orthopedic Surgery, Swedish Neuroscience Institute; 2 Department of Trauma Surgery, BG University Hospital Bergmannsheil, Bochum, Germany; 3 Seattle Science Foundation; 4 Neurological Surgery, Wayne State University; 5 Anatomy, St. George's University; 6 Neurosurgery, Seattle Science Foundation; 7 Anatomy, Seattle Science Foundation; 8 Neurosurgery, Complex Spine, Swedish Neuroscience Institute; 9 Department of Anatomy, St. George's University

**Keywords:** superior mesenteric artery syndrome, pelvis, nutcracker syndrome, pelvic congestion syndrome, superior mesenteric artery

## Abstract

Superior mesenteric artery (SMA) syndrome is a rare clinical entity. We report a female patient presenting with abdominopelvic pain and diagnosed with superior mesenteric artery syndrome. Direct venography revealed a large ovarian varix that was treated with hysterectomy and unilateral oophorectomy. SMA syndrome can have many presentations often with small bowel obstruction. Obstruction of only the ovarian vein with resultant ovarian varix is an unusual presentation.

## Introduction

Superior mesenteric artery (SMA) syndrome is a rare clinical entity that results from vascular compression of the third portion of the duodenum between the angle of the abdominal aorta and the SMA [1]. The syndrome was first described in 1842 by the Austrian professor Carl von Rokitansky, who observed the compression of the third part of the duodenum over the lumbar spine [1-2]. However, the first comprehensive series of 75 patients with SMA syndrome was not published until 1927 by Wilke [1-2]. His name ultimately became a common eponym for superior mesenteric artery syndrome.

The incidence of SMA syndrome in the general population is 0.013-3.0% with a mortality rate of 33% [1, 3-4]. The incidence is higher in young female patients who have had significant weight loss. Other potential causes are surgeries for spinal deformities, a higher than normal insertion of the ligament of Treitz, as well as conditions that lead to a loss of retroperitoneal fat [3-4]. We present an exceptionally rare form of SMA syndrome, which presents with the obstruction of only the ovarian vein with resultant ovarian varix as an unusual presentation. Informed consent was obtained from the patient for this study.

## Case presentation

**Figure 1:**
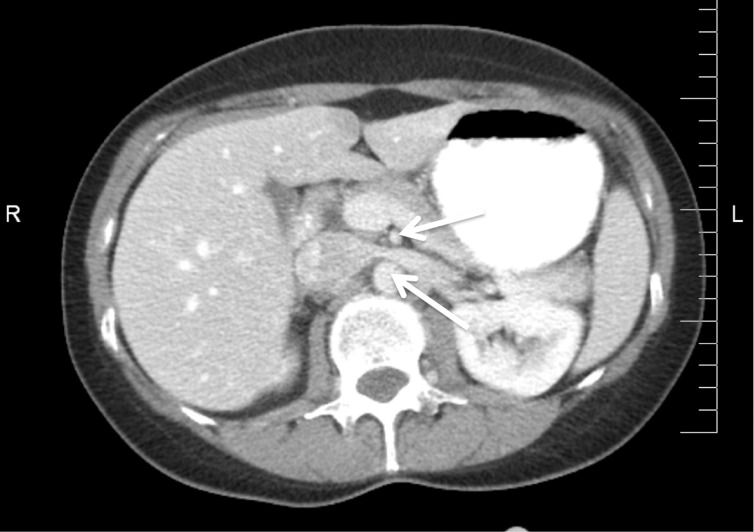
Axial CT of the abdomen Relationship of the superior mesenteric artery (upper arrow) and aorta (lower arrow)

A previously healthy 36-year-old female patient with one previous healthy birth presented with poorly localized abdominopelvic pain lateralized to the left side but vague in nature. The pain was described as being worse in the supine position and after eating. There was no history of hematuria, proteinuria, or bowel obstruction. A physical examination failed to reveal any tenderness to palpation. The peripheral pulses were normal. The radiographs of the abdominopelvic region were normal so was the computed tomography (CT) scan demonstrated in Figure 1.

**Figure 2:**
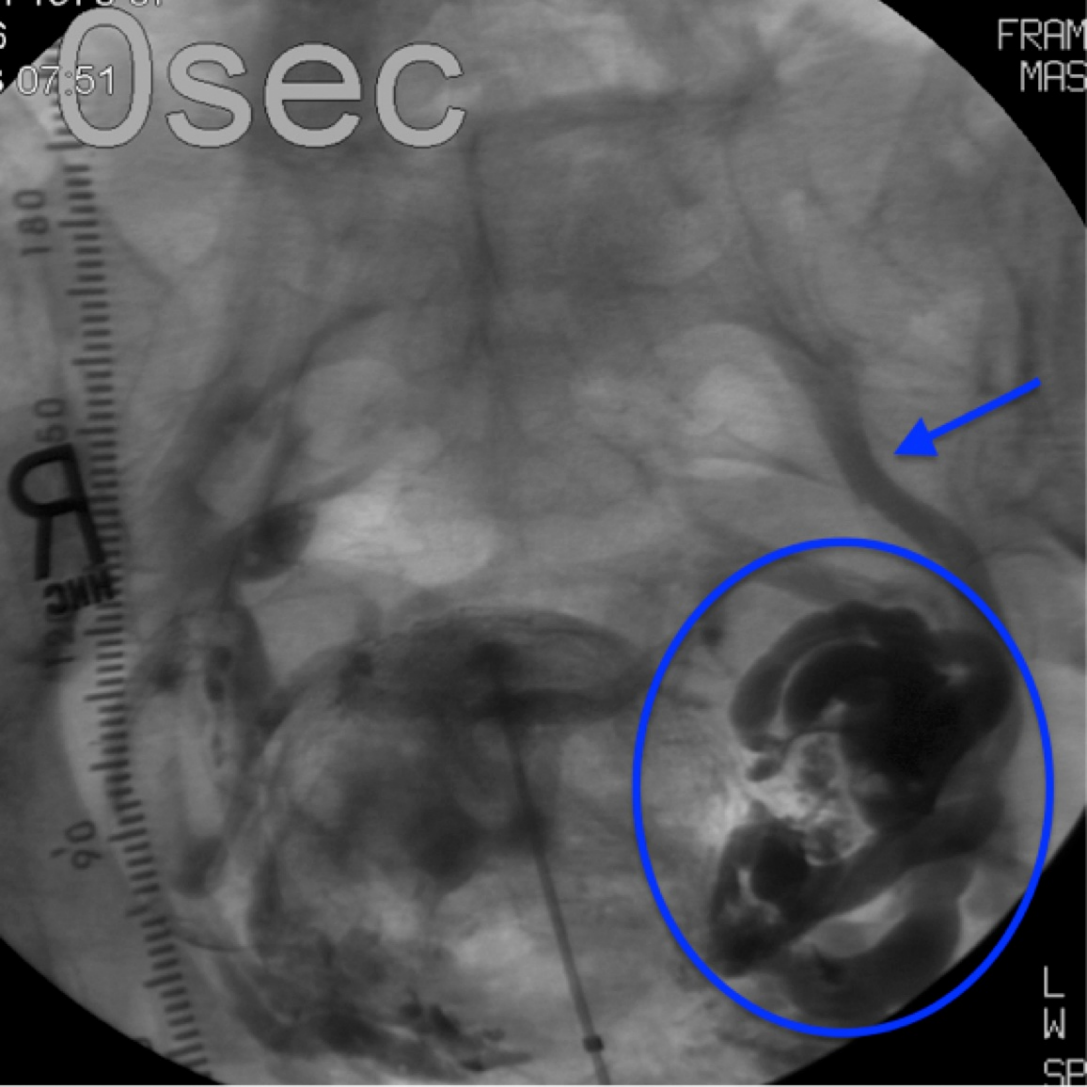
Intrauterine venography Enlarged left ovarian vein (arrow) and huge ovarian varix (within circle)

However, direct uterine injection venography revealed a very large ovarian varix with no proximal flow into the left ovarian vein shown in Figure 2. The diagnosis of SMA syndrome was made and hysterectomy with left side oophorectomy was performed. At the six-month follow-up, the patient’s symptoms had completely resolved.

## Discussion

The SMA usually originates at the level of the L1 vertebra, behind the neck of the pancreas and leaves the aorta at an acute angle [1]. The normal angle that the SMA takes off from the abdominal aorta is at 45 degrees but ranges from 38–56 degrees [2, 5-6], and when the angle becomes hyperacute, ranging from 6–25 degrees as in SMA syndrome [2], it may cause luminal obstruction of the duodenum. The angle between the SMA and the duodenum is maintained by fat and lymphatic tissues, and certain conditions (anorexia nervosa, malabsorption, hypercatabolic states after surgery/burns, congenitally low origin of the SMA) may predispose an individual to SMA syndrome [5-7].

It is the loss of the retroperitoneal fat padding that leads to the acute angulation seen in SMA syndrome [4]. In the adult population, symptoms may not first appear until the angle between the SMA and the aorta falls below 20 degrees, and it is believed to be lower for pediatric patients [3]. The symptoms of SMA syndrome occur secondary to the duodenal obstruction that can present with early satiety, epigastric pain, possibly severe post-prandial pain/fullness, nausea and vomiting that may be bilious, anorexia, eructation, reflux, and 'food fear' leading to malnutrition, weight loss, and poor weight gain [6-7]. Conservative treatment for SMA syndrome mainly consists of weight gain achieved orally or parenterally to reconstitute the mesenteric fat pad [3]. Failure of non-invasive conservative therapy is followed by surgical therapy with duodenojejunostomy being the preferred current treatment [3].

A rare clinical entity that arises as a result of a hyperacute angle between the aorta and the SMA is the nutcracker syndrome in which the left renal vein (LRV) is compressed between the abdominal aorta and the SMA as it crosses the aorta to enter the inferior vena cava (IVC), leading to renal and pelvic congestion [8]. The symptoms of LRV outflow obstruction include hematuria and/or proteinuria due to the rupture of thin-walled varices within the renal collecting system and flank and abdominal pain [9-10]. In addition, symptoms can arise from the secondary obstruction of veins that drain into the LRV, most notably the testicular vein in males and the ovarian and pelvic veins in females.

In males, obstructed outflow of the testicular vein can lead to painful dilatation of the pampiniform venous plexus, a condition known as varicocele [9]. This may occur due to obstructed flow at any point along the venous drainage system, but is commonly due to obstruction of the LRV between the SMA and the aorta as it crosses the aorta to enter the IVC. In women, obstructed LRV outflow can lead to secondary obstruction of veins and subsequent varices with a range of symptoms. The patients may be asymptomatic with incidental pelvic varicosities found on imaging of the pelvis, or they may have symptoms including any of the following: dyspareunia, dysuria, dysmenorrhea, and chronic pelvic pain [8, 10]. The presence of many of the preceding symptoms is often referred to as pelvic congestion syndrome [8].

In our review, the authors describe a female patient with SMA syndrome and huge, symptomatic left ovarian and parauterine varices. These varices were treated with hysterectomy and oophorectomy with vein ligation and resulted in the subsequent resolution of the patient’s symptoms. One very interesting note is that the patient’s LRV was not affected as is commonly seen in the nutcracker syndrome.

## Conclusions

SMA syndrome can have many presentations often with small bowel obstruction. However, obstruction of the ovarian vein with resultant ovarian varix as the only presentation is unusual.
